# Prevalence of Malaria and Associated Factors during the Minor Malaria Season among Febrile Under-Five Children Attending Mohammed Akile Memorial General Hospital

**DOI:** 10.1155/2024/6365077

**Published:** 2024-05-14

**Authors:** Temesgen File, Feysal Jemal

**Affiliations:** ^1^Department of Medical Laboratory Science, Rift Valley University, Adama, Ethiopia; ^2^Department of Public Health, Rift Valley University, Adama, Ethiopia

## Abstract

Malaria is one of the major public health problems in sub-Saharan Africa, including Ethiopia. Children under the age of five are immunologically naive to plasmodium parasites, making them the most vulnerable group to clinical manifestations of malaria infection. Despite the severity of the disease in children under five years of age, most studies on malaria focus on the adult population. In the present study, a cross-sectional study design based on health facilities was used during the minor malaria season from February 18 to May 28, 2023, at Mohammed Akile Memorial General Hospital, in Afar Regional State, district of Amibara located in the town of Berta. The finding revealed that 19.8% prevalence among symptomatic children with 61.04% and 38.96% of *P. falciparum* and *P. vivax*, respectively. In the present study area, malaria infection in children under five years of age is significantly associated with the presence of stagnant water in the residential area, inappropriate or no use of insecticide-treated net, and indoor residual spraying (IRS). The prevalence of malaria among symptomatic children under five years of age is higher compared to the national prevalence of malaria among the general population. Therefore, community mobilization through health promotion, aiming to interrupt the transmission of malaria at the community level, is paramount.

## 1. Introduction

Despite enormous efforts directed towards its control and eventual elimination, malaria has long been a major global public health concern. Sub-Saharan Africa carries a disproportionately high share of the burden. In 2022 alone, the region contributed to 94% and 95% of global malaria cases and deaths, respectively. Children under the age of five accounted for approximately 80% of all malaria deaths in the region [1–3].

About 68% of the Ethiopian population inhabits 75% of the country's landmass, which is a malarious region. In 2018 alone, the number of confirmed malaria cases reported was 1,206,892. Of this, 883,886 (69.2%) and 181,964 (30.8%) were the two coendemic malaria parasites, *P. falciparum* and *P. vivax*, respectively [4]. In Ethiopia, there are two seasons of malaria transmission: the major and minor seasons. The major season of transmission occurs between September and December in most areas, following the main rainy season from June to August. The minor transmission season occurs from April to May, after a short rainy season from February to March [5]. These biannual transmission patterns interfere with the main agricultural seasons, impairing productivity and contributing to poverty.

Children under five years of age, which are immunologically naive to malaria parasites, constitute the most vulnerable group to clinical manifestations during malaria infection [3, 6], leading to severe outcomes in three ways: First, since children do not normally have acquired immunity, they are more likely to have serious infections. Seizures or comas (cerebral malaria) are symptoms of malaria that can cause immediate death. Second is through consequences associated with recurring infections, such as anemia. Finally, it causes low birth weight during pregnancy and increases the probability of death in the first month of life [7], resulting in considerable child death in sub-Saharan Africa (SSA). An estimated 2% of children recovering from cerebral malaria develop learning impairments and disabilities, including epilepsy and spasticity, resulting from brain damage caused by infection [8].

Despite a dramatic reduction in the prevalence of malaria in Ethiopia, the disease remains one of the main public health concerns. Furthermore, the prevalence of malaria infection in children under the age of five in Ethiopia ranges from 16 to 54% [9], contributing 10% of all deaths in children [10].

Several studies were carried out to measure the prevalence rate of malaria among children in various locations in Ethiopia, with widely different and varying findings. The Afar Region is Ethiopia's most lowland and malarious region, where the majority of its population is at risk of infection, particularly under-five children and pregnant women. Furthermore, according to the 2016 Ethiopian Demographic and Health Survey (EDHS) report, the region has the highest mortality rate among children under five years of age compared to other regions (81 deaths per 1000 live births) [11]. Studies also showed that more than one in five children under five years of age were infected with malaria [12]. Therefore, the share of malaria-related deaths in children under five years of age is paramount. Understanding the malaria status of children in a particular study area is, therefore, critical for designing an effective and tailored intervention strategy.

Although the magnitude of malaria infection has been recognized by health facilities as one of the main diseases in the population, there is inadequate data on the magnitude of malaria among febrile under-five children. That could help to evaluate the status of existing interventions. Furthermore, there were inadequate data on under-five malaria in the present study area. Therefore, the study is aimed at examining the prevalence of malaria among under-five symptomatic children attending Mohammed Akile Memorial General Hospital, Afar Regional State, Amibara District, located in the city of Berta.

## 2. Materials and Methods

### 2.1. Study Area and Period

**Figure 1:**
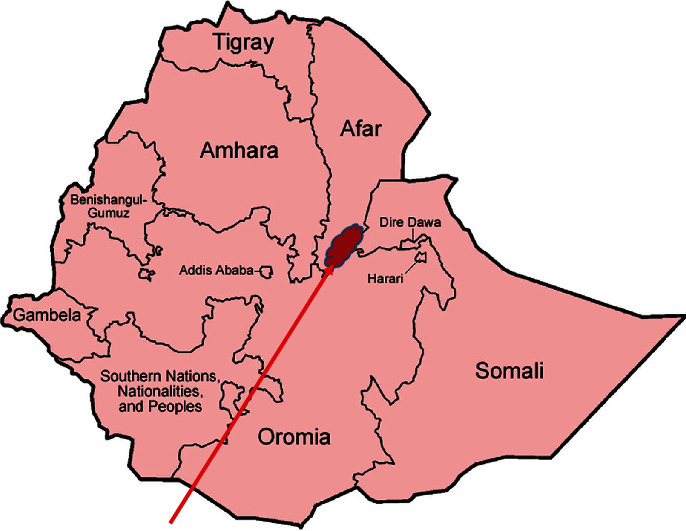
Location map of the Amibara District (Berta City), Afar Regional State, Eastern Ethiopia [15].

A cross-sectional study based on health facilities was conducted from February 18 to May 28, 2023, in children under five years of age (children up to the age of 59 months) to assess the prevalence and associated factors of malaria in symptomatic children under five years of age attending Mohammed Akile Memorial General Hospital in Afar, Ethiopia. The hospital is located in the regional state of Afar, Gabi Rasu (zone 3), district of Amibara, in the city of Berta. The Amibara District is located at 9° 19′43.83′N and 40° 10′51.6′E. The altitudinal range of the study areas lies between 741 and 746 m above sea level. The climate data from the Awash Seven meteorological station were the closest and most representative for both districts. The mean annual temperature for the district was 27°C. The mean minimum and mean maximum annual temperatures for the district were 16.7°C and 37.8°C, respectively, and the mean annual precipitation was 490 mm [13]. Berta town is 353.2 km from the capital and 255.4 km from Samara, the capital city of the regional state. Based on the population census of 2007, the area has more than 300,000 people [14] living in the area of the hospital services in the region. Furthermore, the hospital also serves inhabitants of neighboring districts of Oromia, Amhara, and Somalia (Figure 1).

### 2.2. Study Design and Data Collection

A blood sample from symptomatic malaria patients was collected from febrile under-five children (38°C and above) detected through a mercury thermometer commonly placed under the armpit. The capillary blood sample of the febrile child under five years of age was taken aseptically from a finger prick or big toe using a sterile lancet. The collected blood sample is tested for malaria microscopy and Abbott RDT multispecies conformation tests to detect histidine-rich protein 2 (HRP-2) for *P. falciparum* and lactate dehydrogenase (pLDH) for *P. vivax* following the World Health Organization (WHO) and nationally established malaria protocols [16–18].

Additionally, data from consenting caregivers/mothers of infants with febrile disease was collected from the pediatric outpatient department (OPD) of the hospital, through pretested standardized structured questionnaires by the laboratory technologist. The questionnaire includes interviewer-administered self-report on sociodemographic data, indoor residual spraying (IRS), insecticide-treated net (ITN) utilization, history of fever, rural/urban residence, and availability/unavailability of stagnant water within one kilometer of their locality of parents or caregivers. The questionnaire was first developed in English, later translated into the Afar language, the indigenous local language in the region, and then translated back to English to ensure its consistency by language professionals. Furthermore, the reliability test for the data collection tool was performed, showing the reliability statistics (Cronbach's alpha = 0.564).

### 2.3. Sample Size and Sampling Strategy

A sample size of 389 children was calculated using the population proportion formula [19, 20].

(1) $$\begin{array}{c} \;N=\frac{Z^{2}\;P\left(1-P\right)}{D^{2}}, \end{array}$$

where *N* is the required sample size, *Z* is a standardized normal deviation value that corresponds to the level of statistical significance equal to 1.96, *P* is the estimate of the prevalence of malaria in children under 5 years of age in Afar region, Ethiopia which is 64% [11], *D* = is the error margin that corresponds to the level of precision of the results desired (*α* = 0.05) *N* = ((1.96)^2^ 0.64(1 − 0.64))/((0.05)^2^) = 354 + 10%(non-response rate, to compensate mothers or guardians possibly missed from an interview) = 389.

Since the study was not a community-based survey, the consecutive sampling technique was used in febrile under-five children from pediatric OPD of the hospital who qualify for the inclusion criteria, until the final sample size was reached during the study period. Moreover, seasonal dynamics of malaria transmission during the minor malaria season in Ethiopia showed relatively stable transmission [21], which favors the representativeness of the calculated sample size in the study in focus.

### 2.4. Inclusion Criteria

Symptomatic children who came to the hospital with a history of fever 38°C or above in the last 48 hours and tested for malaria at the hospital at the study site during the study period were included.

### 2.5. Exclusion Criteria

- Febrile under-five children who took antimalaria drugs in the same week and came for follow-up
- Children whose body temperature is less than 38°C, during hospital presentation, and those who had no history of fever in the last 48 hours were excluded from the study
- Children under five years of age whose parents or guardians are unwilling to participate in the study

### 2.6. Data Quality Assurance

Microscopic diagnosis of malaria was made using thick and thin blood films prepared from a finger prick and stained with Giemsa. The quality of Giemsa was checked by positive slides prepared from a positively preserved sample in the laboratory. Furthermore, RDT can also be used for malaria diagnosis following the manufacturer's protocol to ensure a quality malaria diagnosis. Generally, the national standard operational procedure (SOP) for sample collection and malaria microscopic and RDT examination protocol was performed to ensure data quality. The data collection format of each data collector was checked by the second microscopist unaware of the smear result on a daily basis and by one of the principal investigators on a weekly basis.

### 2.7. Data Management and Analysis

All data were entered into EPI Info-7 software and then transferred to SPSS software version 20.0 (SPSS Inc., Chicago, USA) for analysis. Descriptive statistics were used to calculate the frequencies. The results were analyzed using bivariate analysis to provide insight between two categorical variables in the present study. After identifying the variables that significantly affected malaria positivity rate in the bivariate analysis, multiple logistic regression was used to predict the most favorable variables for malaria positivity. And the results were presented using tables. A significance level of *P* < 0.05 was used to assess the statistical significance of accepting or rejecting the hypotheses.

### 2.8. Ethical Considerations

Ethical clearance was obtained from the Adama campus Ethics Review Committee of Rift Valley University (number: RVU/ERD 089/23, dated 01 February 2023) and consent of the local authorities to collect data from Mohammed Akile Memorial General Hospital located in the city of Berta. Permission to collect data from the hospital was obtained from the hospital administration office. Written consent was obtained from the respondents to carry out the research before the questionnaire was administered. Study participants were allowed to ask questions to ensure confidentiality. Participants who did not have an interest were allowed to withdraw from the study. Study participants were convinced that the questionnaires distributed were confidential and used only for academic purposes.

## 3. Result

### 3.1. Characteristics of the Study Population

A total of 389 study participants were included in the present study, with a response rate of 100%. Microscopic and RDT-confirmed malaria cases among febrile under-five children who attended Mohammed Akile Memorial General Hospital during the study period were 77 (19.8%) with a 95% CI (16.2-23.9), of which 47 (61.04%) and 30 (38.96%) were positive for *P. falciparum* and *P. vivax*, respectively. From the total of study participants, children whose ages ranged from 25 to 36 months represented 103 (26.5%) with a mean age of 32 months ± 15.2 SD. Of the total study subjects, 257 (66.1%) were from rural areas (mainly pastoralists). Regarding the educational status of mothers or caregivers, 151 (38.8%) of them were illiterate (never had formal education). Furthermore, bivariate analysis revealed that microscopic and RDT-based detection of malaria cases in the study area did not show significant variation with age, sex, educational status of mother/caregivers, and mother/caregiver history fever before hospital presentation. On the other hand, the use of ITN, IRS use, and the presence of stagnant water in the residential area showed a strong association with malaria cases in the study area (Table 1).

The predictor variables that showed a significant association with dependent variables during the bivariate analysis were selected for multiple regression analysis at 95% confidence intervals. Consequently, when the dependent variable was regressed again in the predictor variables in the multivariate regression analysis, the result overall showed significant association (*X*^2^ (3) = 40.05, *P* < 0.001), revealing that the predictor variables under study significantly influenced the malaria positivity rate, as summarized in Table 2. On the other hand, the effect of individual predictors of ITN use and the presence of stagnant water in the residential area showed a highly significantly influenced malaria positivity rate (*X*^2^ (1) = 45.1, *P* < 0.001). However, the independent role of IRS use has not shown a significant impact (*P* = 0.99) on malaria positivity status when analyzed in multivariate logistic regression.

## 4. Discussion

Malaria in children under five years of age is a significant public health concern, challenging the achievement of the Sustainable Development Goals (SDGs) in SSA. The present study examined microscopic and RDT-based detection of malaria cases and associated factors among febrile under-five children during the minor malaria transmission season in the study area. The finding revealed that of the total 389 symptomatic malaria cases among the target population in the study area, 77 (19.8%) were malaria positive. This is comparable to the Arba Minch report (Southwestern Ethiopia) (22.1%) [18], higher than the South Gondar report (Northwest Ethiopia) (14.6%) [22] and Bena Tsemay District (Southern Ethiopia) (6.1%) [23], and much lower than the study conducted in the Afar Region (Dubti District in Ethiopia) (64%) [10]. This variation could be due to differences in weather conditions, intervention measures, and environmental or behavioral risk factors [24, 25]. Furthermore, the different study periods, target population, and methodologies used might have contributed to variations in prevalence.

The present study also showed that the proportion of the two coendemic malaria parasite species identified in the region was 61.04% and 38.96% for *P. falciparum* and *P. vivax*, respectively. This report is comparable to studies conducted in different parts of Ethiopia [10, 18, 26]. The slight variation could be due to differences in climatic and nonclimatic factors that might have contributed to such variation.

Concerning the relationship between malaria parasite cases and sociodemographic variables, although 43/77 (55.9%) male and 34/77 (44.1%) female malaria patients were identified during the study period, this variation was not statistically significant (*X*^2^ (2) = 2.04, *P* = 0.36) among the study population. On the other hand, reports from SSA countries [26] revealed that the prevalence of malaria cases has increased with age. Consistent with this report, the present study revealed a slight increase in the number of malaria cases in children under five years of age under the age of 48 months. However, similar to the report from Northwestern Ethiopia [27], the present study showed 21/77 (27.2%) of malaria cases from under-five children aged 13 to 24 months, while 11/77 (14.2%) were recorded for under-five children from 49 to 60 months. This could be due to a slight variation in the level of protective immunity among study participants in a malaria-endemic setting. On the other hand, higher (69%) malaria cases were detected in children under five years of age who are symptomatic in rural areas, although the variation is not statistically significant (*P* = 0.56) of malaria cases with locality. This lack of significant urban-rural variation could be due to the low level of urbanization with similar living conditions of the urban area and its rural counterpart of the study area [28]. Analysis of maternal history of fever with malaria cases in the present study area also did not show evidence of association (*P* = 0.083), revealing that the likely cause of fever among mothers or caregivers was not significantly related to malaria cases in children under five years of age in the study area.

Furthermore, the bivariate analysis revealed that the presence of stagnant water near residential areas is significantly (*P* < 0.001) related to malaria cases among children under five years of age in the study area. This could be mainly due to tropical climates, where there is a lot of stagnant water due to flooding, which would favor the breeding of mosquito vectors and eventually malaria transmission in the study area [29, 30]. Similarly, reports from Gondar (Amhara Regional State, Ethiopia) [21], Wogera District (Amhara Regional State, Ethiopia) [31–33], Sherkole District, Benishangul-Gumuz District (Western Ethiopia) [34], and Arba Minch (South Ethiopia) [18] favor the present study. Similarly, in favor of the study in focus, the presence of stagnant water due to flooding favors mosquito breeding and eventually malaria transmission [35–37] in the study area. Likewise, the present study revealed that 68/77 (88%) of the study participants did not properly use ITN or IRS. Thus, bivariate analysis showed that lack or improper use of ITN significantly (*P* < 0.001) contributed to malaria cases in the study subjects in the area. In favor of this, studies have shown that children under five years of age who did not sleep in ITN had a ninefold higher risk of malaria infection compared to those who did sleep in ITN [38–40]. Similarly, children from households without bedspreads are at a higher risk of malaria compared to those who do [39]. This indicates that the use of ITN has been shown to be more effective in the prevention and control of malaria. Furthermore, with increased utilization of indoor residual spraying (IRS), artemisinin-based combination therapy (ACT), and insecticide-treated bed nets (ITNs), malaria deaths and admissions among children under 5 years of age decreased by 81 and 73%, respectively [41].

### 4.1. Limitations of the Study

Although malaria in under-five-year-old children is an important indicator of malaria epidemiology, the present study has a few limitations. (1) The study was conducted only during the minor malaria season, as there was no data during the major malaria season. (2) The present study is also limited to a single health facility-based cross-sectional study. (3) There are also limitations related to malaria microscopy and/or RDT. (4) There is selection bias, due to the convenient sampling technique employed.

## 5. Conclusion and Recommendation

The current study found a higher prevalence of malaria cases (19.8%) among symptomatic children under five years of age, compared to the national prevalence of malaria among symptomatic adults (15.34%) [39]. In the study in focus, the presence of stagnant water in residential areas and inappropriate or no use of ITN/IRS were significantly associated with malaria cases. Therefore, a comprehensive community-based study is recommended that includes both major and minor malaria seasons to depict the real picture of malaria epidemiology among children under five years of age in the study area. The detection of malaria parasites based on microscopic and/or RDT should have been accompanied by molecular detection of the parasites to generate more realistic data. As revealed in the present finding, existing prevention and control measures should also be promoted through health education, targeting not only control and prevention but also the eventual elimination of malaria.

## Figures and Tables

**Table 1:** Summary of data from bivariate analysis of malaria cases among under-five children versus independent variables at Mohammed Akile Memorial General Hospital, Afar, Ethiopia, 2023.

Characteristics	Positive	*P. falciparum*	*P. vivax*	Negative	Total	*P* value
Sex						
Male	43	24	19	312	389	0.36
Female	34	23	11			
Age (in months)						
<12	13	7	6	29	42	0.25
13–24	22	13	9	71	93	
25-36	14	11	3	89	103	
37–48	17	10	7	59	76	
49-60	11	6	5	64	75	
Educational status of mothers/caregivers						
No	29	17	12	122	151	0.19
Primary	37	25	12	107	144	
Secondary	7	3	4	54	61	
Tertiary	4	2	2	29	33	
Residence						
Urban	24	13	11	108	132	0.56
Rural	53	34	19	204	257	
ITN utilization						
Yes		4	5	115	389	<0.001
No	77	43	25	197		
IRS use						
Yes	77	6	3	68	389	0.05
No		41	27	244		
Stagnant water in residential area						
Yes	77	9	8	158	389	<0.001
No		38	22	154		
Mothers/caregiver's history of fever						
Yes	77	46	30	293	389	0.2
No		1	0	20		

**Table 2:** Multiple regression analysis of malaria status in children under five years of age attending Mohammed Akile Memorial General Hospital (*n* = 389) during the study period.

Selected predictor variable	*β*	*X* ^2^	Df	Sig	Exp (*B*)	95% CI for Exp (*B*)	
						Lower	Upper
IRS use	-0.004	45.1	1	0.992	0.996	0.405	2.451
ITN use	1.613		1	0.000	5.018	2.144	11.742
Stagnant water in residential area	-1.399		1	0.000	0.247	0.136	0.449
Overall statistics	-2.4		1	0.000	0.117		

## Data Availability

Data generated or analyzed during this study are included in this manuscript.
